# Supplementary material for: ERECTA Modulates Seed Germination and Fruit Development via Auxin Signaling in Tomato
Source: Int J Mol Sci. 2024 Apr 26;25(9):4754. doi: 10.3390/ijms25094754 (PMC11084166; doi:10.3390/ijms25094754)
Supplement: Supplementary file 1 [file ijms-25-04754-s001.zip › ijms-2950655-supplementary.pdf]

Supplementary Table 1. Primer list used in this research.

Target gene	Primer name	Sequence(5'-3')
For qPCR		
SlUbiquitin	SlUbiquitin-F	CACCAAGCCAAAGAAGATCA
	SlUbiquitin-R	TCAGCATTAGGGCACTCCTT
SlYUC1	YUC1-F	TGGACATTGGTGCATTGGAA
	YUC1-R	AGCCAGTAGCAAGAAGAACAGA
SlYUC3	YUC3-F	TGGCCCTGTAATTGTTGGTG
	YUC3-R	GCCACAGTGATGCGATACAG
SlYUC4	YUC4-F	CGTCCCGACTCGGATTAGAT
	YUC4-R	GCGTTCCAACGTCAAGTACA
SlYUC5	YUC5-F	ATGGCTTGTCGTTGCTACTG
	YUC5-R	CAACCAACGACCACCACTTT
SlYUC6	YUC6-F	TGGCAACATTTGATAATCATGTAGA
	YUC6-R	TGCAGATGTAGCAAGACCAC
SlYUC7	YUC7-F	TGGAGAAATACAAGAAATTGATTG
	YUC7-R	AATCCAACTGCATATAAACCAGC
SlARF2A	ARF2A-F	GTGTCGGAATAGTTGATGCTGATAC
	ARF2A-R	TACACCAGCTCACCCTCTCGC
SlARF2B	ARF2B-F	GCTTGTGACAGTGCCATGTG
	ARF2B-R	TGCTGGTCTGAAGCTTGGTT
SlARF5	ARF5-F	CCTCAGAGTTTGTCATTCCT
	ARF5-R	AACATCATTCCAAATCTCATACC
SlARF6A	ARF6A-F	CCAACATATCCCTAGTACTTCAG
	ARF6A-R	GTGCCTGAGATATTAGTTGGT
SlARF7	ARF7-F	AGACGTTACTCGCTACA
	ARF7-R	ACTTCAGCGCAAGACA
SlARF8B	ARF8B-F	GTCAGTCCGTGATCATAGAG
	ARF8B-R	GGAATCCAAGCTACAATTTCC
SlABI3	ABI3-F	ATCCAAAACTCTGCCCCTTT
	ABI3-R	TAGCACAAGTGCCCAACCT
SlABI5	ABI5-F	GGGAAATGTTTCGTTGGAGA
	ABI5-R	TGTATGTTGCACCCGTTGTT
SlER	ER-F	ACAACAATTTATCAGGCGATG
	ER-R	CTAGAGAAATTATTGCCGGTT

SlERL1	ERL1-F	GCAGTTAAGCGACTTTACACC
	ERL1-R	CAATCTTCAGACGTGTTTCCC
SlIAA9	IAA9-F	TAGATGCTTTACCTGATTACGACA
	IAA9-R	TGCAGACAAACTCCAATATCAAAC
For Sequencing		
SlER	ER436-R	AATGATGCCATGGTAGACTCA
	ER782-R	TACAACACATTCTCCACGTC
	ER1094-F	CACATTATAGACTATGCCTGT
	ER1384-R	CTAACAATCTCGGAAAGCA
	ER1750-F	TTTATTCATATAGGGGACTGC
	ER2402-F	GAGCTGCAATATGTTGAGTGG
	ER2726-F	CATTTGTTTGATTATTTAGCCTT
	ER3269-F	TTATCGAAGTTACCGTCCA
	ER3946-F	AATTTAAGGAGCATCATGGAG
	ER4438-F	ACAACAATTTATCAGGCGATG
	ER4719-F	CTTTTGCTATGTCTCCGAA
	ER5551-F	ATGGCACTTCATGTTTACGAG
	ER6006-F	TGTAGCCCTCGAATAATCCAC
	ER6401-F	TAACTAAGGCAGCAAACGA

Supplementary Table 2. Correspondence table of *YUC* and *FZY* genes analyzed in this research.

YUC	gene ID	*FZY*
YUC1	Solyc06g008050	*FZY4*
YUC2	Solyc06g065630	*FZY1*
YUC3	Solyc06g083700	*FZY5*
YUC4	Solyc08g068160	*FZY2*
YUC6	Solyc09g074430	*FZY6*
YUC7	Solyc09g091090	*FZY3*

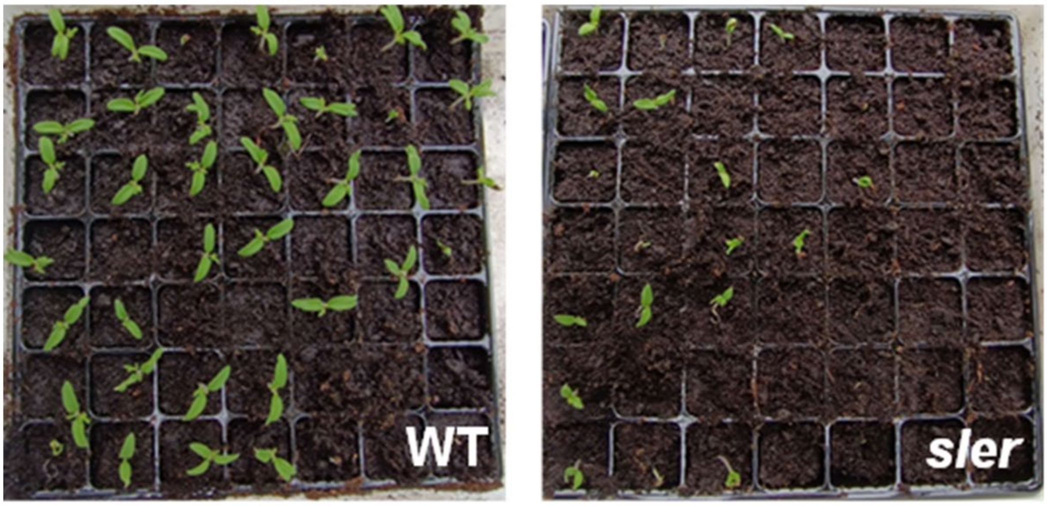

Supplementary Figure 1. The state of seed germination in the soil. The germination rates of WT and *sler* seeds were 75.5% and 38.8% respectively.

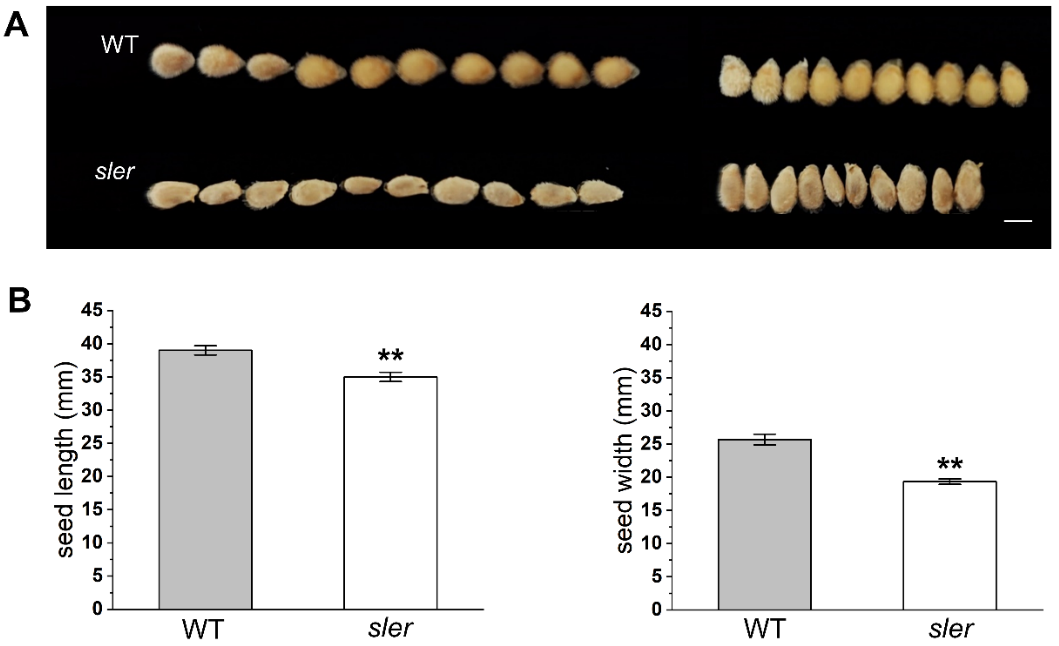

Supplementary Figure 2. Seed size of Micro-Tom WT and *sler*. Seed fullness of WT and *sler* (A), bar=2 mm. Seed length and width (B). Ten seeds were considered as one biological replicate. Error bars represent the standard deviations. Significant differences were determined by two-tailed Student's *t* test (**, 0.001<P < 0.01), n=3.

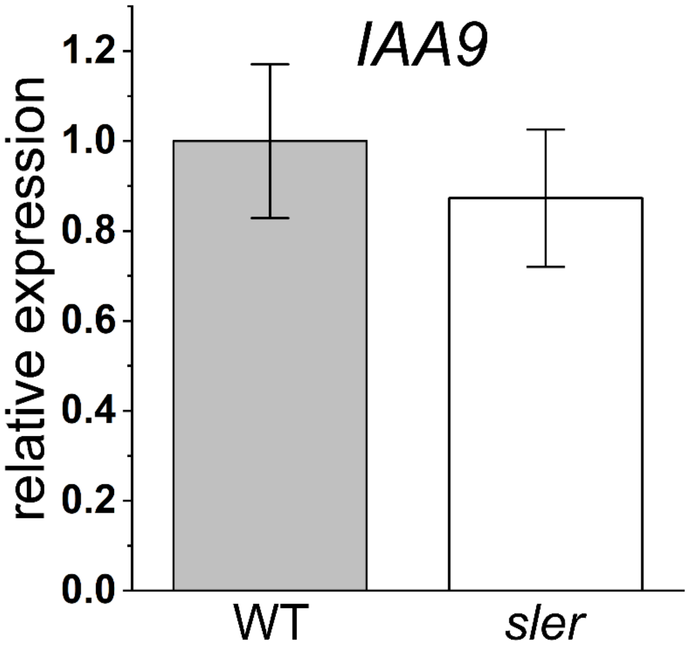

Supplementary Figure 3. Relative expression of *IAA9* between WT and *sler* ovary in DAF0. Error bars represent the standard deviations. Significant differences were determined by two-tailed Student’s *t* test. n=3.

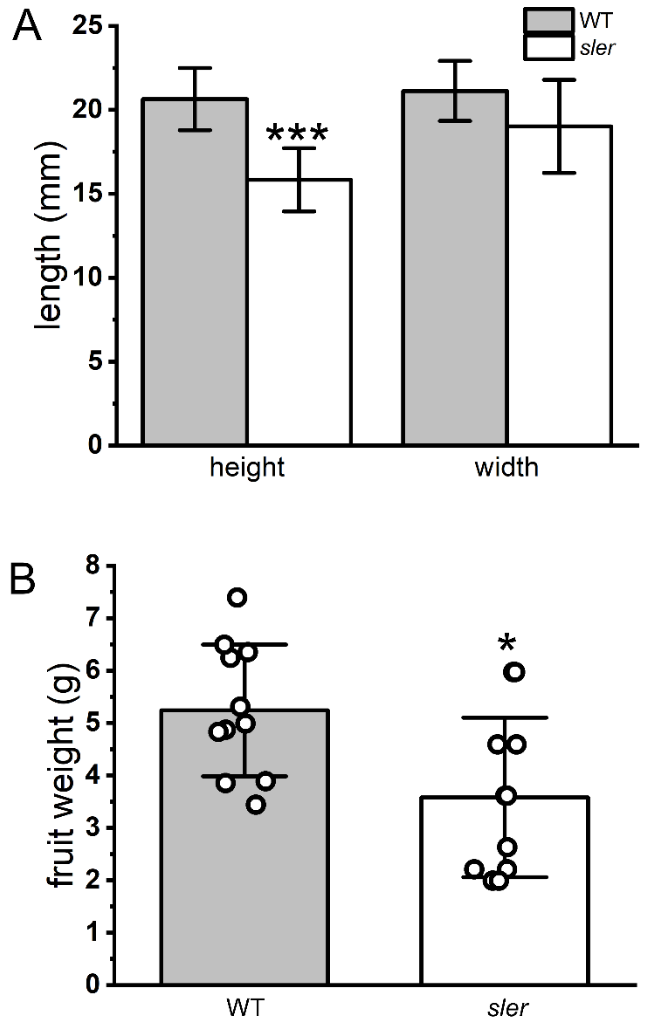

Supplementary Figure 4. Fruit size and weight of WT and *sler*. Fruit height and width of WT and *sler* (A). Fruit weight of WT and sler (B). Error bars represent the standard deviations. Significant differences were determined by two-tailed Student's *t* test(*,P < 0.05; ***, P < 0.001). n=10.
